# Effect of Right Ventricular Outflow Tract Material Properties on Simulated Transcatheter Pulmonary Placement

**Published:** 2026-01-08

**Authors:** Jalaj Maheshwari, Wensi Wu, Christopher N. Zelonis, Steve A. Maas, Kyle Sunderland, Yuval Barak-Corren, Stephen Ching, Patricia Sabin, Andras Lasso, Matthew J. Gillespie, Jeffrey A. Weiss, Matthew A. Jolley

**Affiliations:** a Department of Anesthesiology and Critical Care Medicine, Children’s Hospital of Philadelphia, Philadelphia, 19104, PA, USA; b Cardiovascular Institute, Children’s Hospital of Philadelphia, Philadelphia, 19104, PA, USA; c Department of Mechanical Engineering and Applied Mechanics, University of Pennsylvania, Philadelphia, 19104, PA, USA; d Scientific Computing and Imaging Institute, University of Utah, Salt Lake City, UT, USA; e Laboratory for Percutaneous Surgery, Queen’s University, Kingston, ON, Canada; f Division of Pediatric Cardiology, Children’s Hospital of Philadelphia, Philadelphia, 19104, PA, USA

**Keywords:** transcatheter pulmonary valve deployment, heterogeneous tissue properties, polynomial chaos expansion, sensitivity analysis

## Abstract

Finite element (FE) simulations emulating transcatheter pulmonary valve (TPV) system deployment in patient-specific right ventricular outflow tracts (RVOT) assume material properties for the RVOT and adjacent tissues. Sensitivity of the deployment to variation in RVOT material properties is unknown. Moreover, the effect of a transannular patch stiffness and location on simulated TPV deployment has not been explored. A sensitivity analysis on the material properties of a patient-specific RVOT during TPV deployment, modeled as an uncoupled HGO material, was conducted using FEBioUncertainSCI. Further, the effects of a transannular patch during TPV deployment were analyzed by considering two patch locations and four patch stiffnesses. Visualization of results and quantification were performed using custom metrics implemented in SlicerHeart and FEBio. Sensitivity analysis revealed that the shear modulus of the ground matrix (c), fiber modulus $\left(k_{1}\right)$, and fiber mean orientation angle (γ) had the greatest effect on 95th %ile stress, whereas only c had the greatest effect on 95th %ile Lagrangian strain. First-order sensitivity indices contributed the greatest to the total-order sensitivity indices. Simulations using a transannular patch revealed that peak stress and strain were dependent on patch location. As stiffness of the patch increased, greater stress was observed at the interface connecting the patch to the RVOT, and stress in the patch itself increased while strain decreased. The total enclosed volume by the TPV device remained unchanged across all simulated patch cases. This study highlights that while uncertainties in tissue material properties and patch locations may influence functional outcomes, FE simulations provide a reliable framework for evaluating these outcomes in TPVR.

## 2. INTRODUCTION

Pulmonary insufficiency is a consequence of surgical repair in Tetralogy of Fallot (ToF), leading to right ventricular dilation, ventricular arrhythmias, and heart failure. Pulmonary valve replacement (PVR), traditionally with a surgically implanted bioprosthetic valve, is often required to prevent many of these long-term complications [1, 2, 3, 4]. In the small subset of ToF patients who had a cylindrical RVOT conduit placed as part of their initial repair, a balloon-expandable transcatheter PVR (TPVR) has been used without the need for cardiopulmonary bypass [5, 6, 7, 8, 9, 10]. However, the vast majority of patients with ToF undergo surgical repair using a transpulmonary annulus patch, resulting in a dilated and heterogeneous shape of the native RVOT. As a result, balloon-expandable transcatheter valves cannot be safely used in over 75 percent of ToF patients in need of PVR [11, 12, 13, 14, 15, 16, 17].

The limited applicability of balloon-expandable valves within these complex outflow tracts has stimulated the development of self-expanding valve platforms designed to conform to a variety of native RVOT shapes and sizes [18, 19, 20]. These self-expanding TPVR systems can conform to a wide range of heterogeneous native RVOT shapes, but precise matching of the optimal TPV to an individual patient is required [18, 21, 22]. However, as the number of available devices for TPVR continues to increase, along with the number of potential candidates, the ability to screen patients efficiently and choose an appropriate device becomes critical [22, 13, 23, 24].

Current screening techniques to identify suitable candidates are labor-intensive and continue to rely primarily on 2D measurements from CT reconstructions of the RVOT and occasional implantation of actual devices into 3D-printed models to visually assess device fit [24]. While several studies have explored transcatheter aortic valve implantation (TAVI) [25, 26, 27, 28, 29], computational simulation studies examining TPVR are sparse [17, 30, 31, 32]. While additional finite element (FE) simulations to inform matching of devices to an image-derived patient-specific anatomy are emerging [33], all simulations assume material properties for the RVOT within the mediastinum based on very limited data[34, 35]. Further, the material properties of a surgically altered right ventricular outflow tract could vary from those of normal tissue. For example, the effect of patch material in the RVOT, which may alter tissue local stiffness and compliance at the time of initial repair on simulated TPV deployment, has not yet been investigated. Unfortunately, it is not currently possible to extract patient-specific material properties of the RVOT and pulmonary artery, and the sensitivity of the simulated deployment of self-expanding TPV to variations in RVOT tissue properties, and the effect of incorporation of heterogeneous materials into the reconstructed RVOT remains unknown. As such, there is a critical need to understand the sensitivity of emerging FE simulations of TPVR to both variation of RVOT material properties and the heterogeneity of material properties throughout the RVOT.

In the present work, we performed a two-stage sensitivity analysis using our open-source simulation framework implemented within SlicerHeart [36] and FEBio [37] to better understand how uncertainties in tissue material parameters affect the simulated results of TPV deployment. First, we assessed the sensitivity of RVOT stress and strain distributions following TPV deployment to uncertainty in tissue properties, assuming uniform properties throughout the RVOT. Subsequently, we investigated the influence of nonuniform tissue properties introduced by the presence of a transannular patch. In particular, we examined how variations in patch stiffness relative to native tissue and its anatomical placement affect the simulated RVOT biomechanics of TPV deployment. We performed both a traditional parameter exploration as well as a polynomial chaos expansion (PCE)-based uncertainty quantification analysis using UncertainSCI [38] to quantify these effects.

## 3. METHODS

### 3.1. Procuring RVOT Model and Segmentation

Patients with a diagnosis of ToF who underwent TPVR and for whom computer tomography angiography (CTA) of the RVOT prior to TPVR had been previously acquired were identified from an existing institutional database. A patient with a typical geometry for the RVOT was chosen as an example. The Institutional Review Board at the Children’s Hospital of Philadelphia approved this study.

CTA images were acquired on a dual-source scanner (Siemens Healthcare, Forchheim, Germany) using a retrospective ECG-gating technique (2 × 128 × 0.6-mm slice collimation). Low-osmolar iodinated contrast (Iohexol, OmnipaqueTM, 350 mg/mL, GE Healthcare Inc.) was injected via peripheral intravenous access with a dose of 2 mL/kg (up to 100 mL) for the patient. These retrospectively EKG-gated acquired images were typically reconstructed choosing the 30% phase for systole and 90% for diastole.

CTA images were then imported into 3D Slicer (www.slicer.org) in Digital Imaging and Communication in Medicine (DICOM) format. CT images of the TPV25 device were acquired on a CT scanner and segmented to create templates for mesh creation 3D CAD software (Fusion 360, Autodesk, San Francisco, USA). The dimensions of the TPV device were also confirmed using digital calipers. Segmentation of the RVOT used in this study was created using a deep learning-based module in 3D Slicer as used in previous research [33, 39].

### 3.2. Computational Model Setup

The inner surface of the RVOT vessel was exported as a shell mesh from 3D Slicer as a stereolithography (.STL) file. This .STL file was imported into Blender (V4.4), and the RVOT surface was remeshed as a quad-dominant shell mesh with an average mesh size of 1.5 mm to reduce the mesh density. The quad-meshed surface was exported from Blender in a .ply format and imported into FEBio. To create a solid mesh for the RVOT, the shell mesh was then extruded outward, normal to each surface element to have a thickness of 1.5 mm [32, 40].

To capture the anisotropic and hyperelastic material behavior and account for the fiber orientations in the vessel, the RVOT was assigned an uncoupled Holzapfel-Gasser-Ogden (HGO) constitutive material model [41, 42]. The deviatoric and volumetric behavior for this material model is controlled by the strain-energy function:

(1) $${\text{Ψ}}_{r}={\tilde{\text{Ψ}}}_{r}(\tilde{C})+U(J),$$

where,

(2) $${\tilde{\text{Ψ}}}_{r}=\frac{c}{2}\left({\tilde{I}}_{1}-3\right)+\frac{k_{1}}{2k_{2}}\sum_{\alpha =1}^{2}\left(\exp\left(k_{2}{\langle {\tilde{E}}_{\alpha }\rangle }^{2}\right)-1\right),$$

and the volumetric strain energy function is:

(3) $$U(J)=\frac{k}{2}\left(\frac{J^{2}-1}{2}-\ln J\right).$$

Here, $\tilde{C}$ is the right Cauchy-Green deformation tensor, ${\tilde{I}}_{1}=tr\tilde{C}$, ${\tilde{I}}_{4\alpha }=a_{\alpha r}\cdot \tilde{C}\cdot a_{\alpha r}$, and α=1,2, and $E_{\alpha }=\kappa \left({\tilde{I}}_{1}-3\right)+(1-3\kappa )\left({\tilde{I}}_{4\alpha }-1\right)$ represents the fiber strain. The input parameters for the HGO material included the material density (ρ), shear modulus of the ground matrix (c), fiber modulus $\left(k_{1}\right)$, fiber exponential coefficient $\left(k_{2}\right)$, fiber mean orientation angle (γ), fiber dispersion (κ), and bulk modulus $\left(k\right)$. The HGO model parameters were defined as per previously published work [31] and are included in Table 1.

The TPV device was modeled using 1-dimensional beam elements. The beams had a circular cross-sectional area with a diameter of 0.375 mm. Input parameters for beam elements included the material density per unit length (ρ), the cross-sectional area of the beam (A), the shear-corrected cross-sectional areas $\left(A_{1},A_{2}\right)$, Young’s modulus (E), shear modulus (G), and the second moments of inertia $\left(I_{1},I_{2}\right)$. Cross-sectional area $\left(\pi r^{2}\right)$ and moments of inertia $\left(\frac{1}{4}\pi r^{4}\right)$ corresponding to a circle were used. Material properties for Nitinol [43] were used to model the TPV device and are included in Table 1.

To uniformly compress the TPV25 device before expanding it in the RVOT, a curved tube covering the TPV25 device with its center along the vessel centerline was used. The tube radius was 28 mm, which was just large enough to contain the TPV device completely without having it penetrate the tube. The tube was split into two parts, allowing for a staged release of the TPV device: first expansion at the distal half, and second expansion at the proximal half of the device. The tube surface was modeled using a triangular shell mesh with a thickness of 2 mm. A neo-Hookean material model was used for the tubes [44], the inputs for which were the material density (ρ), Young’s modulus (E), and Poisson’s ratio (ν). Parameter values for the tube are also included in Table 1.

The distal and proximal ends of the RVOT were fixed. A zero displacement boundary condition was applied to the central nodes of the TPV25 device to prevent it from sliding in the Z direction or vertically along the vessel. The deployment of the TPV device in the RVOT was simulated in three stages: 1) compression of the device, 2) expansion of the device at the distal end, and 3) expansion of the device at the proximal end. The compression and expansion of the device were controlled by applying a normal displacement to the surrounding tube to mimic the self-expanding behavior of the TPV device. Edge-to-surface contact was defined between the TPV device and the tube for all stages, and between the TPV device and the RVOT vessel in the expansion stages. This entire setup process was also previously implemented in our emerging open-source framework [33].

### 3.3. Analysis Setup

#### 3.3.1. Mesh convergence

A mesh convergence analysis was conducted to determine the optimum mesh count for the RVOT. The RVOT wall was meshed with one through six layers of solid mesh. The intramural strain at the distal and proximal locations where the TPV device impinged the RVOT wall, and the 95th%ile and 99th%ile strains in the entire vessel when the stent expands completely were analyzed to determine the appropriate mesh density to use for subsequent analyses.

#### 3.3.2. Sensitivity and uncertainty analysis for RVOT material

A sensitivity analysis was conducted to understand the effect of the HGO material model coefficients $\left(c,k_{1},k_{2},\gamma ,\kappa \right)$ on the TPV device deployment inside the RVOT. A Python subroutine called FEBioUncertainSCI, which interfaces UncertainSCI [38] and the FEBio solver code [37] (www.febio.org), and which has been used in other sensitivity analyses of valve material properties [45, 46], was used. For all HGO material parameters explored, baseline material properties from prior literature exploring RVOTs and self-expanding stents were chosen [31], which are also reported in Table 1.

To find the range of values to use for each HGO material parameter for the sensitivity analysis, adult artery data were used to obtain the standard deviation value of the artery’s material properties from values reported for different arterial layers [47]. Since the media layer is known to dominate the mechanical behavior of arteries [48, 49], values for only the media layer were used. The standard deviation reported as a percentage of the mean for different arterial layers as per [47] is shown in Table 2A.

Using the mean values for parameters as those reported by [31] and the standard deviation percentages from [47], a gamma distribution was determined for each material parameter to obtain the shape (k) and scale factor (θ) to use as inputs for UncertainSCI’s gamma distribution function. Since [31] used κ=1/3, which represents isotropic fiber dispersion, and using any standard deviation value with a gamma distribution would cause a violation of the constraints for κ(0≤κ≤1/3), the mean value for κ from [47] was used. Final mean values and standard deviation percentages for each HGO material parameter are shown in Table 2B.

Obtained values for shape and scale factor for each HGO material parameter for their respective gamma distributions are shown in Table 2C. All HGO material input parameters were varied simultaneously to generate 136 combinations of materials using a fourth-order polynomial chaos expansion (PCE) function. Simulations were conducted for each of the 136 combinations of HGO material parameter values. The 95th%ile, 75th%ile, and mean 1st principal stress and Lagrangian strain of the RVOT, and total- and first-order Sobol indices for each material parameter were extracted.

#### 3.3.3. Transannular patch stiffness

**Figure 1:**
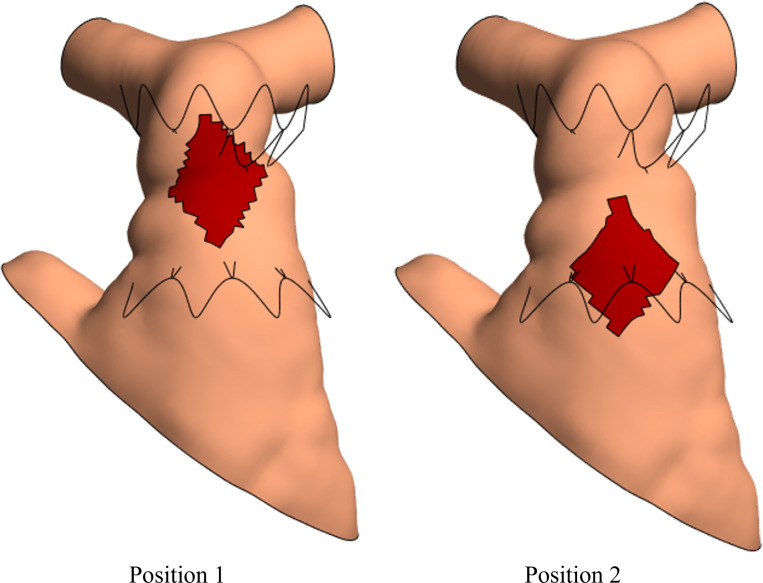
Transannular patch embedded in the RVOT vessel wall at two locations, a distal (position 1) and proximal (position 2) location with respect to the undeformed TPV device.

To understand the effect of a transannular patch on the TPV device deployment in the RVOT, a diamond-shaped patch was embedded in the RVOT. Based on the largest size reported in prior literature [50], the transannular patch measuring 30 mm x 20 mm along its longer and shorter diagonals was modeled and located at two locations where the TPV device interacts with the RVOT vessel wall, a distal position and a proximal position, shown in Figure 1.

The transannular patch was modeled as an isotropic elastic material [51] with material properties based on prior literature [52, 53], which have been highlighted in Table 3. The stiffness of the patch was increased 2x, 4x, and 8x by scaling the Young’s modulus from values reported in prior literature, i.e., 1.1×10^3^ kPa. The maximum, 95th%ile, 75th%ile, and mean first principal stresses and Lagrangian strains were analyzed.

## 4. RESULTS

### 4.1. Mesh Convergence Analysis

**Figure 2:**
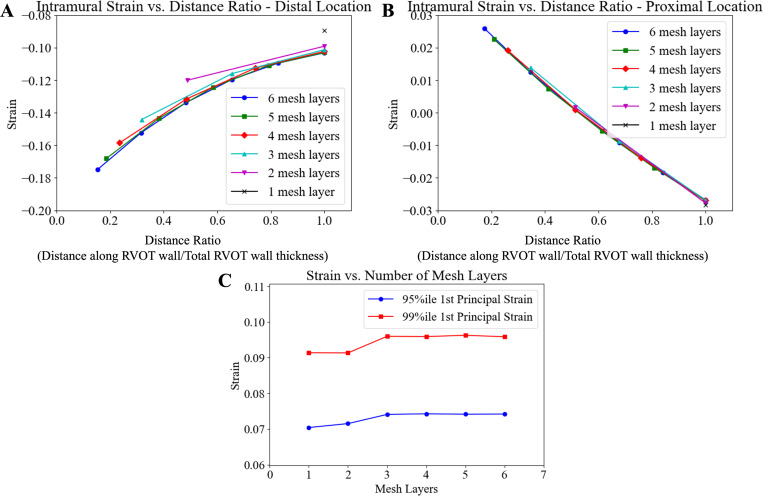
Mesh convergence analysis. (**A**) intramural strain vs. distance ratio at a distal location and (**B**) at a proximal location where the TPV device impinges the RVOT wall. (**C**) 95th%ile and 99th%ile strains in the entire RVOT at complete TPV device expansion. Based on the mesh convergence plots, the model containing 4 mesh layers in the RVOT wall was chosen for the subsequent simulations.

To determine the optimal mesh size for the RVOT, a mesh convergence analysis was conducted. Figure 2A and 2B show the intramural strain along the RVOT wall for different numbers of mesh layers (one through six) at the distal location and proximal location on the RVOT wall, respectively. In these figures, the x-axis represents the distance ratio or the distance along the vessel wall with respect to the total thickness of the RVOT wall (1.5 mm), and the y-axis represents the strain along the RVOT vessel wall. Figure 2C represents the 95th and 99th%ile 1st principal strains in the entire RVOT vessel for the different mesh layers across the vessel wall. The 95th and 99th%ile strains were chosen to eliminate the effect of any ‘hotspots’ where the TPV device impinged on the vessel wall. From these figures, the intramural strains and 1st principal strains converged at 4 mesh layers through the RVOT vessel wall. Therefore, for all subsequent simulations and analyses, a 4-layered mesh RVOT wall FE model was used.

### 4.2. Sensitivity and Uncertainty Analysis

Of the 136 total simulations generated for the sensitivity analysis, 133 ran to completion successfully and 3 failed. The failed simulations had HGO material parameters that situated it on the lower extremity of the gamma distribution curves for each parameter, resulting in a highly distensible and unstable RVOT wall. Results were compiled for the remaining successful 133 simulations.

**Figure 3:**
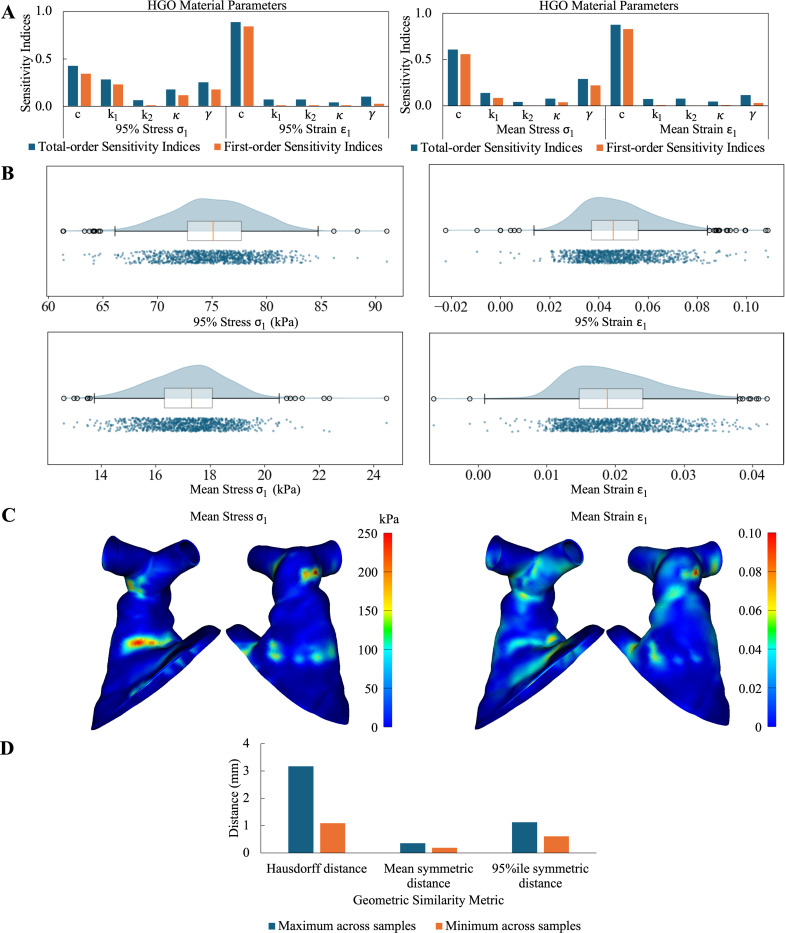
(**A**) Total and first-order sensitivity indices for the HGO material parameters for 95%ile and mean 1st principal stress and Lagrangian strain in the RVOT. (**B**) Raincloud plot showing the distribution of 1000 sampling combinations of HGO material parameters for 95th%ile and mean stress and strain in the RVOT vessel wall at maximum TPV device expansion. (**C**) Mean 1st principal stress and Lagrangian strain distribution across all simulations conducted for sensitivity analysis. (**D**) Geometric comparison metrics calculated across the 133 completed sensitivity simulations using a baseline simulation with vessel material parameters defined as per [31, 32]. Plot depicts the maximum and minimum values across all samples.

The total and first-order sensitivity indices at the 95th%ile and mean stress and Lagrangian strain are shown in Figure 3A. Within the sampling space, the shear modulus of the ground matrix (c), fiber modulus $\left(k_{1}\right)$, and fiber mean orientation angle (γ) had the greatest influence on 95th%ile stress, while $k_{1}$ followed by γ had the greatest influence on 75th%ile and mean stress in the RVOT vessel wall. The shear modulus of the ground matrix (c) also had the greatest influence on the 95th%ile, 75th%ile, and mean Lagrangian strain. Both total and first-order sensitivity indices followed similar patterns, with the first-order indices contributing the greatest to the total-order indices.

The input HGO material parameters and the corresponding output were used to train a PCE emulator as previously explored [45, 46]. 1000 sampling combinations of the HGO material parameters were queried from the PCE emulator. Figure 3B depicts the raincloud plots showing the minimum, maximum, and median values, and the distribution of these 1000 combinations on the output metrics. High uncertainties were observed for all plotted metrics, i.e. 95th%ile and mean stresses and strains in the entire RVOT model, indicating that the RVOT material is highly sensitive to the input material parameter values. Furthermore, the variation in each output metric was large, greater than 10% of the difference in the minimum and maximum values.

Figure 3C depicts the mean 1st principal stress and Lagrangian strain calculated across all simulations run for the sensitivity analysis to obtain potential ‘hot-spot’ regions of high stress and strain. Regions of highest stress and strain, irrespective of the RVOT wall material parameters, were observed at the location of the proximal and distal ends of the stent, where the stent wireframe contacts the RVOT wall. Using the simulation with parameter values from [31, 32] as a baseline, similarity between the RVOT geometries at complete device expansion was determined by computing the Hausdorff distance, mean symmetric distance, and 95%ile symmetric distance for the 133 completed simulations. Figure 3D highlights the maximum and minimum metric values across the 133 simulations. The maximum and minimum Hausdorff distances were 10.5% and 3.6% of the perimeter-derived diameter at the narrowest region of the baseline simulation RVOT at complete stent expansion. These metric values highlight that while stress and strain values varied across the generated samples, stress and strain patterns and RVOT geometries were fairly similar on complete stent expansion.

### 4.3. Transannular Patch Stiffness Analysis

**Figure 4:**
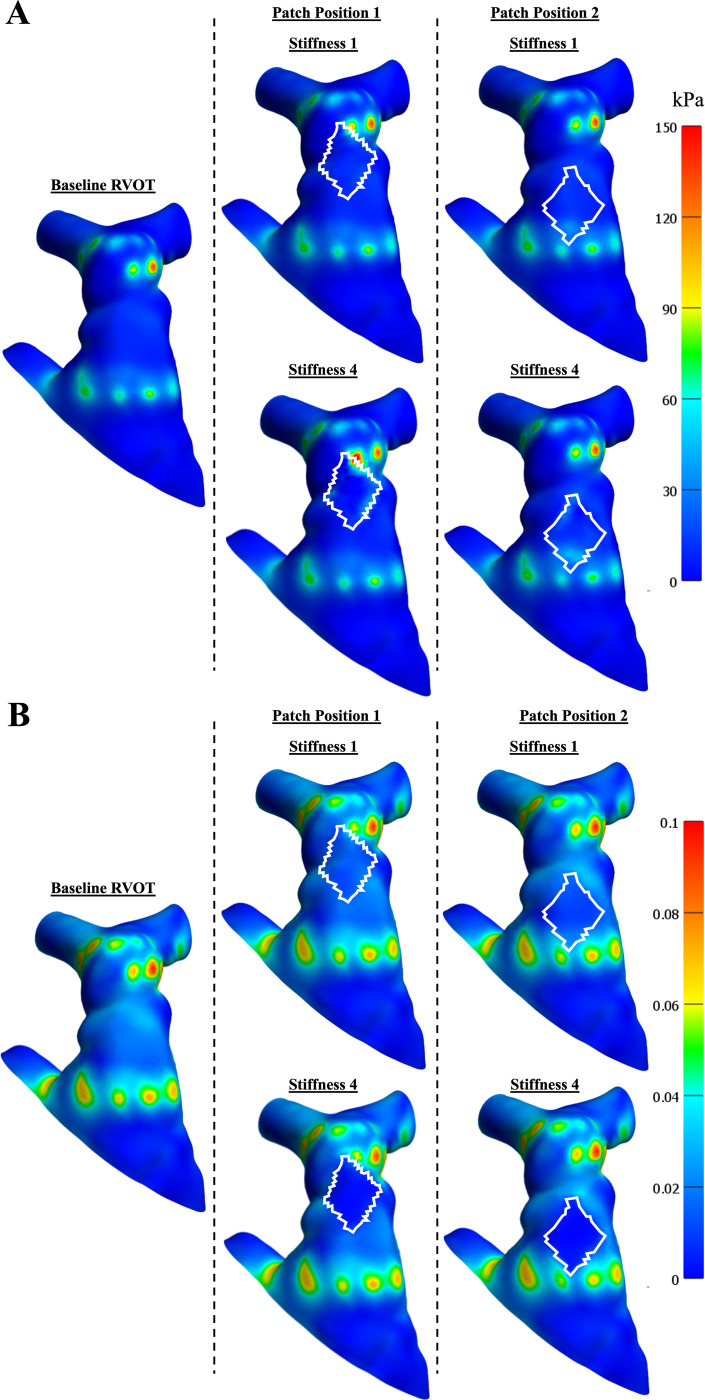
(**A**) 1st principal stress and (**B**) 1st principal Lagrangian strain distribution across baseline, patch position 1, and patch position 2 simulations for stiffness 1 and stiffness 4 conditions.

Figure 4 depicts the 1st principal stress and Lagrangian strain distribution for a baseline RVOT without a transannular patch, a patch in position 1, and a patch in position 2 for two stiffness values. Due to the presence of the patch, greater regions of stress are observed at the boundary where the patch attaches to the RVOT. Conversely, the strain observed in the patched region was minimal compared to that observed in the rest of the RVOT. Higher regions of stress and stress were also observed at the locations where the proximal and distal ends of the stent impinge on the RVOT wall. Additionally, as the stiffness of the patch increased, stress at the boundary of the patch and RVOT increased, while strain decreased.

**Figure 5:**
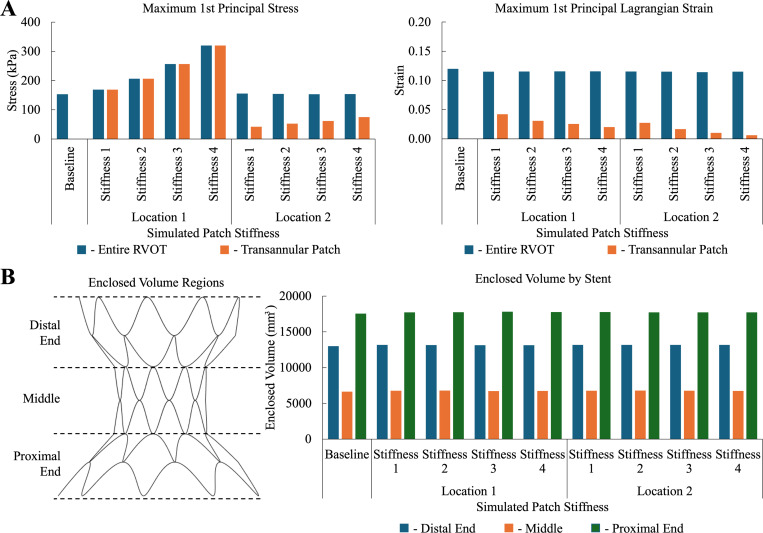
(**A**) 1st principal stress and 1st principal Lagrangian strain distribution across baseline, patch position 1, and patch position 2 simulations in the entire RVOT and only the transannular patch. (**B**) Enclosed volume by the stent across simulated conditions, separated by the distal region of the stent, the middle region of the stent, and the proximal region of the stent.

Figure 5A highlights the maximum 1st principal stress and Lagrangian strain for all patch simulation cases in the entire RVOT and just the transannular patch. For patch position 1, the maximum stress was observed in the patched region. For patch position 2, maximum stress in the RVOT was observed in a location different from the patched region. Maximum strain in the entire RVOT was observed away from the patched region and was similar across all simulated conditions. Across both patched locations, maximum stress in the transannular patch increased, whereas maximum strain decreased as patch stiffness increased. Maximum stress and strain for the same patch stiffness values were greater in patch position 1 than in position 2. Additionally, the 95th%ile, 75th%ile, and mean first principal stresses and Lagrangian strains followed the same trends as maximum stress and Lagrangian strain.

The volume enclosed by the stent was calculated by dividing the stent into three regions: the distal end, the middle, and the proximal end, and is shown in Figure 5B. Across all simulated conditions, the variation in the enclosed volume across these three regions was minimal.

## 5. DISCUSSION

We conducted a sensitivity analysis of the effect of RVOT material properties and material property spatial heterogeneity on the simulation of a self-expanding TPV deployment in the RVOT of a patient with ToF as a foundational step toward using simulations of TPVR to aid the assessment of patient candidacy and optimal device selection in patients with TPVR [22, 24]. We found that primary vessel material properties significantly affect the resulting simulation, such as local stress and strain, but have a relatively lesser effect on overall vessel geometry. Spatial heterogeneity (such as a stiff patch) had less effect on the resulting device conformation but significant effects on local vessel stress.

Simulation of self-expanding transcatheter valves has evolved over the last two decades and is now beginning to be clinically applied to adult TAVR. However, the first transcatheter valve deployed clinically was developed to address pulmonary regurgitation in patients with dysfunctional conduits in congenital heart disease, such as ToF [54]. Self-expanding devices such as the Harmony and Alterra pre-stent system now potentially meet the needs of the largest population with pulmonary insufficiency, represented by patients who have native outflow tracts after transannular patch placement in infancy. In addition, multiple other devices and sizes of devices are in development [20]. However, the new challenge is selecting which of these devices is optimal for an individual patient [22, 24].

Tissue mechanical properties can greatly influence the accuracy of simulations of self-explanding TPVR. Unfortunately, to date, experimentally-derived tissue material data for arteries in biaxial tensile testing has been limited to a small sample of patients, either infants [34] or older adults [47]. Due to difficulty in the characterization of in vivo mechanical properties and the variability of the RVOT among different patients, it is important to understand the sensitivity of the constitutive material model parameters on the mechanical outcome metrics, such as stress and strain due to stent implantation, which may ultimately affect the clinical viability of the stent in a particular patient. To quantify the uncertainty in the simulation outcome, we performed a robust PCE-based uncertainty quantification analysis using UncertainSCI. Our sensitivity analysis revealed that the 95th%ile stress was sensitive to the shear modulus of the ground matrix parameter c, the fiber modulus $k_{1}$, and the fiber mean orientation angle γ, whereas 75th%ile and mean stress were sensitive to c and γ. 95th%ile, 75th%ile, and mean strain were only sensitive to c. Additionally, the first-order sensitivity indices had the greatest effect on total sensitivity indices, indicating that while important, secondary and higher-order indices do not have a significant enough effect on outcome metrics [55]. Despite variability in material properties, the greatest stress and strain regions were consistently at the locations where the proximal and distal ends of the TPV device impinge on the RVOT wall. Vessel material properties influenced the stress and strain values, but their effect on stress and strain distribution across the entire vessel and the expanded vessel geometry was minimal.

Additional material variability that can be introduced in the RVOT wall includes the presence of prosthetic material introduced at the time of transannular patch during the initial surgical operation in infancy. On analyzing the effect of stent deployment in an RVOT with varying patch material stiffness, we found that the regions of greatest stress and least strain were at the junction where the patch embeds in the RVOT wall, irrespective of stiffness. Additionally, increasing the stiffness of the patch increased maximum stress and decreased maximum strain in the patch. However, both the presence of a patch, as compared to a native non-patched RVOT, and the location of the patch had a considerable effect on maximum stress observed in the entire RVOT.

When the patch was located around the distal end of the stent, maximum stress in the entire RVOT was observed in the transannular patch. When the patch was located around the proximal end of the stent, maximum stress was observed away from the patch in a different region of the RVOT. The presence of a transannular patch or the location of the patch did not influence the volume enclosed by the stent. These significant variations highlight the importance of using simulation-based prepositional assessment to inform clinicians.

There are a few limitations to this study. Firstly, data on HGO material constants used in this study, which have been determined through either uniaxial [35] or biaxial tensile testing [34] of pulmonary arteries, are limited to a small sample of patients in a wide age range. The samples of pulmonary arteries tested in these studies correspond to infants aged 8 months or adults aged 44–47 years. TPVR is generally performed for patients across a wide age range, which includes children, adolescents, young adults, and older adults [56, 57, 58]. Therefore, there is a clinical need to evaluate tissue material properties for these age ranges. Constrained by the availability of appropriate tissue samples, future studies on determining elastic properties using 4D-CT-derived tissue deformation data can be a potential avenue to improve the fidelity of simulation results [59]. Secondly, the simulations conducted in this study evaluate biomechanical outcomes across conditions and do not consider blood flow or hemodynamics. Future work will leverage fluid-structure interaction to examine hemodynamic changes of TPV deployment, as has previously been conducted for TAVI [60, 61, 62, 63]. Thirdly, the RVOT model and simulations in this study only considered the diastolic phase of the cardiac cycle. Future work will incorporate both the systolic and diastolic phases to provide a comprehensive assessment over an entire cardiac cycle. Finally, the simulated conditions need to be validated with clinical TPVR procedure geometries. Future work will aim to include this validation to improve predicted outcomes.

## 6. CONCLUSION

FE simulation provides a promising approach for evaluating optimal TPV placements before implantation and improving the success of repair. However, patient-specific tissue properties of the RVOT, which are critical for determining simulation outcomes, are often unknown. To better understand this limitation, we performed a sensitivity study to investigate the effects of material parameters on stress and strain in the RVOT model during TPV deployment. We found that RVOT stress and strain were most sensitive to uncertainties in the shear modulus of the ground matrix. While stress and strain values varied, their distribution across the vessel was similar. Furthermore, the vessel geometry on complete stent expansion was minimally affected by the material parameters. We further investigated model sensitivity for stress, strain, and RVOT enclosed volume in models with a transannular patch. Our results indicate that patch location and stiffness had a substantial influence on both stress and strain values in the RVOT, while changes in enclosed volume were negligible. These collective findings suggest that although the location and material properties of the transannular patch may confound analysis of maladaptive tissue remodeling, FE simulation remains a reliable framework for evaluating immediate functional outcomes in TPVR.

## Figures and Tables

**Table 1:** Material parameters for simulation components.

Material Parameter	Value
**RVOT**	

ρ	1.02×10^−6^ kg/mm3
c	200 kPa
$k_{1}$	13480 kPa
$k_{2}$	1.06
γ	18.85 deg
κ	0.33
k	1500 kPa

**TPV25**	

ρ	6.5×10^−6^ kg/mm^3^
E	4.0×10^7^ kPa
G	1.5×10^7^ kPa
$A=A_{1}=A_{2}$	0.111045 mm^2^
$I_{1}=I_{2}$	0.000970722

**Tube**	

ρ	6.5×10^−6^ kg/mm^3^
E	4.0×10^7^ kPa
ν	0.33

**Table 2:** (**A**) Standard deviation as a percentage of the mean for different arterial layers as per [47]. (**B**) Mean and standard deviation of HGO material parameters used to calculate gamma distribution parameters. (**C**) Shape and scale parameters for gamma distributions for each HGO material parameter.

A	Arterial Layer	Metric	c	$k_{1}$	$k_{2}$	γ	κ	epsilon	Average
	Media	mean	1.27	21.6	8.21	20.61	0.25	0.05	
		std	0.63	7.12	3.27	5.5	0.09	0.02	
		percentage	49.61	32.96	39.83	26.69	36	48.89	39

B	Material Parameter	Mean Value	Standard Deviation (%)
	c	200 kPa	49.61
	$k_{1}$	13480 kPa	32.96
	$k_{2}$	1.06	39.83
	κ	0.0833	36
	γ	18.85 deg	26.69

C	Gamma Distribution Parameters	c	$k_{1}$	$k_{2}$	γ	κ
	Shape (k)	4.0631	9.2050	6.3035	14.0379	7.7160
	Scale (θ)	49.2230	1464.4154	0.1682	1.3428	0.0108

**Table 3:** Material parameter values for transannular patch conditions.

Transannular Patch Stiffness Condition	Density (kg/mm^3^)	Poisson’s Ratio	Young’s Modulus (kPa)
Stiffness 1	1.41×10^−6^	0.495	1.1 ×10^3^
Stiffness 2			2.2 ×10^3^
Stiffness 3			4.4 ×10^3^
Stiffness 4			8.8 ×10^3^

## 1. ABBREVIATIONS

2D: Two Dimensional
3D: Three Dimensional
CTA: Computed Tomography Angiography
DICOM: Digital Imaging and Communications in Medicine
FE: Finite Element
HGO: Holzapfel Gasser Ogden
RVOT: Right Ventricular Outflow Tract
STL: Stereolithography
ToF: Tetralogy of Fallot
TPV: Transcatheter Pulmonary Valve
PVR: Pulmonary Valve Replacement
TPVR: Transcatheter Pulmonary Valve Replacement
TAVI: Transcatheter Aortic Valve Implantation

## References

[1] Geva Tal. Repaired tetralogy of Fallot: the roles of cardiovascular magnetic resonance in evaluating pathophysiology and for pulmonary valve replacement decision support. Journal of Cardiovascular Magnetic Resonance, 13(1):9, January 2011. 21251297 10.1186/1532-429X-13-9 PMC3036629

[2] Lee Cheul, Choi Eun Seok, and Lee Chang-Ha. Long-term outcomes of pulmonary valve replacement in patients with repaired tetralogy of Fallot. European Journal of Cardio-Thoracic Surgery, 58(2):246–252, August 2020. 32047919 10.1093/ejcts/ezaa030

[3] Bokma Jouke P., Geva Tal, Sleeper Lynn A., Lee Ji Hae, Lu Minmin, Sompolinsky Tehila, Babu-Narayan Sonya V., Wald Rachel M., Mulder Barbara J.M., and Valente Anne Marie. Improved Outcomes After Pulmonary Valve Replacement in Repaired Tetralogy of Fallot. Journal of the American College of Cardiology, 81(21):2075–2085, May 2023. 37225360 10.1016/j.jacc.2023.02.052

[4] Geva Tal, Wald Rachel M., Bucholz Emily, Cnota James F., McElhinney Doff B., Mercer-Rosa Laura M., Mery Carlos M., Miles Andrea Leann, Moore Jeremy, and on behalf of the American Heart Association Council on Lifelong Congenital Heart Disease and Heart Health in the Young; Council on Cardiovascular Surgery and Anesthesia; Council on Clinical Cardiology; and Council on Cardiovascular and Stroke Nursing. Long-Term Management of Right Ventricular Outflow Tract Dysfunction in Repaired Tetralogy of Fallot: A Scientific Statement From the American Heart Association. Circulation, 150(25), December 2024. 10.1161/CIR.0000000000001291 39569497

[5] Biernacka Elżbieta Katarzyna, Rużyłło Witold, Demkow Marcin, Kowalski Mirosław, Śpiewak Mateusz, Piotrowski Walerian, Kuśmierczyk Mariusz, Banaś Sławomir, Różanski Jacek, and Hoffman Piotr. Transcatheter pulmonary valve implantation in patients with right ventricular outflow tract dysfunction: early and mid-term results. The Journal of Invasive Cardiology, 27(6):E82–89, June 2015. 26028663

[6] Demkow Marcin, Rużyłło Witold, Biernacka Elżbieta Katarzyna, Kalińczuk Łukasz, Śpiewak Mateusz, Kowalski Mirosław, Sitkowska Ewa, Kuśmierczyk Mariusz, Różanski Jacek, Banaś Sławomir, Chmielak Zbigniew, and Hoffman Piotr. Percutaneous edwards SAPIEN^™^ valve implantation for significant pulmonary regurgitation after previous surgical repair with a right ventricular outflow patch. Catheterization and Cardiovascular Interventions, 83(3):474–481, February 2014. Publisher: Wiley. 23804542 10.1002/ccd.25096

[7] Ghawi Hani, Kenny Damien, and Hijazi Ziyad M.. Transcatheter Pulmonary Valve Replacement. Cardiology and Therapy, 1(1), December 2012. Publisher: Springer Science and Business Media LLC. 10.1007/s40119-012-0005-9 PMC4107445 25135159

[8] Kenny Damien, Hijazi Ziyad M., Kar Saibal, Rhodes John, Mullen Michael, Makkar Raj, Shirali Girish, Fogel Mark, Fahey John, Heitschmidt Mary G., and Cain Christopher. Percutaneous Implantation of the Edwards SAPIEN Transcatheter Heart Valve for Conduit Failure in the Pulmonary Position. Journal of the American College of Cardiology, 58(21):2248–2256, November 2011. Publisher: Elsevier BV. 22078433 10.1016/j.jacc.2011.07.040

[9] O’Byrne Michael L., Glatz Andrew C., Mercer-Rosa Laura, Gillespie Matthew J., Dori Yoav, Goldmuntz Elizabeth, Kawut Steven, and Rome Jonathan J.. Trends in Pulmonary Valve Replacement in Children and Adults With Tetralogy of Fallot. The American Journal of Cardiology, 115(1):118–124, January 2015. Publisher: Elsevier BV. 25456860 10.1016/j.amjcard.2014.09.054 PMC4262614

[10] Valente Anne Marie, Gauvreau Kimberlee, Assenza Gabriele Egidy, Babu-Narayan Sonya V, Schreier Jenna, Gatzoulis Michael A, Groenink Maarten, Inuzuka Ryo, Kilner Philip J, Koyak Zeliha, Landzberg Michael J, Mulder Barbara, Powell Andrew J, Wald Rachel, and Geva Tal. Contemporary predictors of death and sustained ventricular tachycardia in patients with repaired tetralogy of Fallot enrolled in the INDICATOR cohort. Heart, 100(3):247–253, February 2014. Publisher: BMJ. 24179163 10.1136/heartjnl-2013-304958 PMC3913216

[11] Gillespie Matthew J., McElhinney Doff B., Jones Thomas K., Levi Daniel S., Asnes Jeremy, Gray Robert G., Cabalka Allison K., Fujimoto Kazuto, Qureshi Athar M., Justino Henri, Bergersen Lisa, Benson Lee N., Haugan, Boe Brian A., and Cheatham John P.. 1-Year Outcomes in a Pooled Cohort of Harmony Transcatheter Pulmonary Valve Clinical Trial Participants. JACC: Cardiovascular Interventions, 16(15):1917–1928, August 2023. 37278682 10.1016/j.jcin.2023.03.002

[12] Gartenberg Ari J., Gillespie Matthew J., and Glatz Andrew C.. Transcatheter Approaches to Pulmonary Valve Replacement in Congenital Heart Disease: Revolutionizing the Management of RVOT Dysfunction? Seminars in Thoracic and Cardiovascular Surgery, 35(2):333–338, 2023. 35259489 10.1053/j.semtcvs.2022.02.009

[13] Jolley Matthew A., Lasso Andras, Nam Hannah H., Dinh Patrick V., Scanlan Adam B., Nguyen Alex V., Ilina Anna, Morray Brian, Glatz Andrew C., McGowan Francis X., Whitehead Kevin, Dori Yoav, Gorman Joseph H., Gorman Robert C., Fichtinger Gabor, and Gillespie Matthew J.. Toward predictive modeling of catheter-based pulmonary valve replacement into native right ventricular outflow tracts. Catheterization and Cardiovascular Interventions, 93(3), February 2019. Publisher: Wiley. 10.1002/ccd.27962 PMC7713792 30444053

[14] Kenny Damien P. and Hijazi Ziyad M.. Current Status and Future Potential of Transcatheter Interventions in Congenital Heart Disease. Circulation Research, 120(6):1015–1026, March 2017. 28302745 10.1161/CIRCRESAHA.116.309185

[15] Patel Neil D., Levi Daniel S., Cheatham John P., Qureshi Shakeel A., Shahanavaz Shabana, and Zahn Evan M.. Transcatheter Pulmonary Valve Replacement: A Review of Current Valve Technologies. Journal of the Society for Cardiovascular Angiography & Interventions, 1(6):100452, November 2022. 39132347 10.1016/j.jscai.2022.100452 PMC11307711

[16] Schievano Silvia, Coats Louise, Migliavacca Francesco, Norman Wendy, Frigiola Alessandra, Deanfield John, Bonhoeffer Philipp, and Taylor Andrew M.. Variations in right ventricular outflow tract morphology following repair of congenital heart disease: implications for percutaneous pulmonary valve implantation. Journal of Cardiovascular Magnetic Resonance: Official Journal of the Society for Cardiovascular Magnetic Resonance, 9(4):687–695, 2007. 17578725 10.1080/10976640601187596

[17] Capelli Claudio, Taylor Andrew M., Migliavacca Francesco, Bonhoeffer Philipp, and Schievano Silvia. Patient-specific reconstructed anatomies and computer simulations are fundamental for selecting medical device treatment: application to a new percutaneous pulmonary valve. Philosophical Transactions of the Royal Society A: Mathematical, Physical and Engineering Sciences, 368(1921):3027–3038, June 2010. 10.1098/rsta.2010.0088 PMC2944396 20478919

[18] Benson Lee N., Gillespie Matthew J., Bergersen Lisa, Cheatham Sharon L., Hor Kan N., Horlick Eric M., Weng Shicheng, McHenry Brian T., Osten Mark D., Powell Andrew J., and Cheatham John P.. Three-Year Outcomes From the Harmony Native Outflow Tract Early Feasibility Study. Circulation. Cardiovascular Interventions, 13(1):e008320, January 2020. 32525412 10.1161/CIRCINTERVENTIONS.119.008320

[19] Zahn Evan M., Chang Jennifer C., Armer Dustin, and Garg Ruchira. First human implant of the Alterra Adaptive PrestentTM : A new self-expanding device designed to remodel the right ventricular outflow tract. Catheterization and Cardiovascular Interventions: Official Journal of the Society for Cardiac Angiography & Interventions, 91(6):1125–1129, May 2018. 29521437 10.1002/ccd.27581 PMC5969108

[20] Jin Qinchun, Long Yuliang, Zhang Gejun, Pan Xin, Chen Mao, Feng Yuan, Liu Jinfen, Yu Shiqiang, Pan Wenzhi, Zhou Daxin, and Ge Junbo. Five-year follow-up after percutaneous pulmonary valve implantation using the Venus P-valve system for patients with pulmonary regurgitation and an enlarged native right ventricular outflow tract. Catheterization and Cardiovascular Interventions: Official Journal of the Society for Cardiac Angiography & Interventions, 103(2):359–366, February 2024. 38054354 10.1002/ccd.30916

[21] Schoonbeek Rosanne C., Takebayashi Satoshi, Aoki Chikashi, Shimaoka Toru, Harris Matthew A., Fu Gregory L., Kim Timothy S., Dori Yoav, McGarvey Jeremy, Litt Harold, Bouma Wobbe, Zsido Gerald, Glatz Andrew C., Rome Jonathan J., Gorman Robert C., Gorman Joseph H., and Gillespie Matthew J.. Implantation of the Medtronic Harmony Transcatheter Pulmonary Valve Improves Right Ventricular Size and Function in an Ovine Model of Postoperative Chronic Pulmonary Insufficiency. Circulation: Cardiovascular Interventions, 9(10), October 2016. Publisher: Ovid Technologies (Wolters Kluwer Health). 10.1161/CIRCINTERVENTIONS.116.003920 PMC6563325 27662847

[22] Gillespie Matthew J., Benson Lee N., Bergersen Lisa, Bacha Emile A., Cheatham Sharon L., Crean Andrew M., Eicken Andreas, Ewert Peter, Geva Tal, Hellenbrand William E., Hor Kan N., Horlick Eric M., Jones Thomas K., Mayer John, McHenry Brian T., Osten Mark D., Powell Andrew J., Zahn Evan M., and Cheatham John P.. Patient Selection Process for the Harmony Transcatheter Pulmonary Valve Early Feasibility Study. The American Journal of Cardiology, 120(8):1387–1392, October 2017. 28823485 10.1016/j.amjcard.2017.07.034

[23] Bergersen Lisa, Benson Lee N., Gillespie Matthew J., Cheatham Sharon L., Crean Andrew M., Hor Kan N., Horlick Eric M., Lung Te-Hsin, McHenry Brian T., Osten Mark D., Powell Andrew J., and Cheatham John P.. Harmony Feasibility Trial. JACC: Cardiovascular Interventions, 10(17):1763–1773, September 2017. 28882284 10.1016/j.jcin.2017.05.034

[24] McElhinney Doff B., Gillespie Matthew J., Aboulhosn Jamil A., Cabalka Allison K., Morray Brian H., Balzer David T., Qureshi Athar M., Hoskoppal Arvind K., and Goldstein Bryan H.. Transcatheter Pulmonary Valve Replacement With the Harmony Valve in Patients Who Do Not Meet Recommended Oversizing Criteria on the Screening Perimeter Plot. Circulation. Cardiovascular Interventions, 17(5):e013889, May 2024. 38606564 10.1161/CIRCINTERVENTIONS.123.013889

[25] Auricchio F., Conti M., Morganti S., and Reali A.. Simulation of transcatheter aortic valve implantation: a patient-specific finite element approach. Computer Methods in Biomechanics and Biomedical Engineering, 17(12):1347–1357, September 2014. 23402555 10.1080/10255842.2012.746676

[26] Morganti S., Conti M., Aiello M., Valentini A., Mazzola A., Reali A., and Auricchio F.. Simulation of transcatheter aortic valve implantation through patient-specific finite element analysis: Two clinical cases. Journal of Biomechanics, 47(11):2547–2555, August 2014. 24998989 10.1016/j.jbiomech.2014.06.007

[27] Ovcharenko E.A., Klyshnikov K.U., Yuzhalin A.E., Savrasov G.V., Kokov A.N., Batranin A.V., Ganyukov V.I., and Kudryavtseva Y.A.. Modeling of transcatheter aortic valve replacement: Patient specific vs general approaches based on finite element analysis. Computers in Biology and Medicine, 69:29–36, February 2016. 26708469 10.1016/j.compbiomed.2015.12.001

[28] Sturla Francesco, Ronzoni Mattia, Vitali Mattia, Dimasi Annalisa, Vismara Riccardo, Preston-Maher Georgia, Burriesci Gaetano, Votta Emiliano, and Redaelli Alberto. Impact of different aortic valve calcification patterns on the outcome of transcatheter aortic valve implantation: A finite element study. Journal of Biomechanics, 49(12):2520–2530, August 2016. 27059259 10.1016/j.jbiomech.2016.03.036 PMC5038160

[29] Bosi Giorgia M., Capelli Claudio, Cheang Mun Hong, Delahunty Nicola, Mullen Michael, Taylor Andrew M., and Schievano Silvia. Population-specific material properties of the implantation site for transcatheter aortic valve replacement finite element simulations. Journal of Biomechanics, 71:236–244, April 2018. 29482928 10.1016/j.jbiomech.2018.02.017 PMC5889787

[30] Bosi Giorgia M., Capelli Claudio, Khambadkone Sachin, Taylor Andrew M., and Schievano Silvia. Patient-specific finite element models to support clinical decisions: A lesson learnt from a case study of percutaneous pulmonary valve implantation. Catheterization and Cardiovascular Interventions, 86(6):1120–1130, November 2015. 25855063 10.1002/ccd.25944

[31] Donahue Carly L., Aggarwal Varun, and Barocas Victor H.. Finite Element Modeling Using Patient-Specific Geometry to Predict Aortic Valve Insufficiency During Percutaneous Pulmonary Valve Implantation. In 2022 Design of Medical Devices Conference, page V001T02A001, Minneapolis, MN, USA, April 2022. American Society of Mechanical Engineers.

[32] Donahue Carly L., Westman Claire L., Faanes Brittany L., Qureshi Athar M., Barocas Victor H., and Aggarwal Varun. Finite element modeling with patient-specific geometry to assess clinical risks of percutaneous pulmonary valve implantation. Catheterization and Cardiovascular Interventions, 103(6):924–933, May 2024. 38597297 10.1002/ccd.31016

[33] Zelonis Christopher N., Maheshwari Jalaj, Wu Wensi, Maas Steve A., Aslan Seda, Sunderland Kyle, Ching Stephen, Koluda Ashley, Barak-Corren Yuval, Mangine Nicolas, Sabin Patricia M., Lasso Andras, Laurence Devin W., Herz Christian, Gillespie Matthew J., Weiss Jeffrey A., and Jolley Matthew A.. Integrated Open-Source Framework for Simulation of Transcatheter Pulmonary Valves in Native Right Ventricular Outflow Tracts, 2025. Version Number: 2.

[34] Cabrera M.S., Oomens C.W.J., Bouten C.V.C., Bogers A.J.J.C., Hoerstrup S.P., and Baaijens F.P.T.. Mechanical analysis of ovine and pediatric pulmonary artery for heart valve stent design. Journal of Biomechanics, 46(12):2075–2081, August 2013. Publisher: Elsevier BV. 23849135 10.1016/j.jbiomech.2013.04.020

[35] Jia Yueqian, Qiao Yangyang, Argueta-Morales I. Ricardo, Maung Aung, Norfleet Jack, Bai Yuanli, Divo Eduardo, Kassab Alain J., and DeCampli William M.. Experimental Study of Anisotropic Stress/Strain Relationships of Aortic and Pulmonary Artery Homografts and Synthetic Vascular Grafts. Journal of Biomechanical Engineering, 139(10), October 2017. Publisher: ASME International. 10.1115/1.4037400 28753691

[36] Lasso Andras, Herz Christian, Nam Hannah, Cianciulli Alana, Pieper Steve, Drouin Simon, Pinter Csaba, St-Onge Samuelle, Vigil Chad, Ching Stephen, Sunderland Kyle, Fichtinger Gabor, Kikinis Ron, and Jolley Matthew A.. SlicerHeart: An open-source computing platform for cardiac image analysis and modeling. Frontiers in Cardiovascular Medicine, 9, September 2022. Publisher: Frontiers Media SA. 10.3389/fcvm.2022.886549 PMC9485637 36148054

[37] Maas Steve A., Ellis Benjamin J., Ateshian Gerard A., and Weiss Jeffrey A.. FEBio: Finite Elements for Biomechanics. Journal of Biomechanical Engineering, 134(1), January 2012. Publisher: ASME International. 10.1115/1.4005694 PMC3705975 22482660

[38] Burk Kyle M., Narayan Akil, and Orr Joseph A.. Efficient sampling for polynomial chaos-based uncertainty quantification and sensitivity analysis using weighted approximate Fekete points. International Journal for Numerical Methods in Biomedical Engineering, 36(11), November 2020. Publisher: Wiley. 10.1002/cnm.3395 PMC8138745 32794272

[39] Diaz-Pinto Andres, Alle Sachidanand, Nath Vishwesh, Tang Yucheng, Ihsani Alvin, Asad Muhammad, Pérez-García Fernando, Mehta Pritesh, Li Wenqi, Flores Mona, Roth Holger R., Vercauteren Tom, Xu Daguang, Dogra Prerna, Ourselin Sebastien, Feng Andrew, and Cardoso M. Jorge. MONAI Label: A framework for AI-assisted interactive labeling of 3D medical images. Medical Image Analysis, 95:103207, July 2024. 38776843 10.1016/j.media.2024.103207

[40] Vanderveken Emma, Vastmans Julie, Claus Piet, Verbeken Eric, Fehervary Heleen, Van Hoof Lucas, Vandendriessche Katrien, Verbrugghe Peter, Famaey Nele, and Rega Filip. Mechano-biological adaptation of the pulmonary artery exposed to systemic conditions. Scientific Reports, 10(1):2724, February 2020. 32066803 10.1038/s41598-020-59554-7 PMC7026065

[41] Christian Gasser T., Ogden Ray W, and Holzapfel Gerhard A. Hyperelastic modelling of arterial layers with distributed collagen fibre orientations. Journal of The Royal Society Interface, 3(6):15–35, February 2006. 16849214 10.1098/rsif.2005.0073 PMC1618483

[42] Maas Steve A., Ateshian Gerard A., Weiss Jeffrey A., and Herron Michael. Uncoupled Holzapfel-Gasser-Ogden, March 2025.

[43] Vemury Chandra Mouli, Corradi Marco, Abozaid Feras, Charles Alasdair, and Hughes David. The behaviour of Nitinol Wire Bundles for Structural Applications. Recent Progress in Materials, 03(01):1–1, August 2019.

[44] Maas Steve A., Ateshian Gerard A., Weiss Jeffrey A., and Herron Michael. Neo-Hookean, March 2025.

[45] Wu Wensi, Ching Stephen, Maas Steve A., Lasso Andras, Sabin Patricia, Weiss Jeffrey A., and Jolley Matthew A.. A Computational Framework for Atrioventricular Valve Modeling Using Open-Source Software. Journal of Biomechanical Engineering, 144(10), October 2022. Publisher: ASME International. 10.1115/1.4054485 PMC9254695 35510823

[46] Wu Wensi, Ching Stephen, Sabin Patricia, Laurence Devin W., Maas Steve A., Lasso Andras, Weiss Jeffrey A., and Jolley Matthew A.. The effects of leaflet material properties on the simulated function of regurgitant mitral valves. Journal of the Mechanical Behavior of Biomedical Materials, 142:105858, June 2023. Publisher: Elsevier BV. 37099920 10.1016/j.jmbbm.2023.105858 PMC10199327

[47] Holzapfel Gerhard A., Sommer Gerhard, Gasser Christian T., and Regitnig Peter. Determination of layer-specific mechanical properties of human coronary arteries with nonatherosclerotic intimal thickening and related constitutive modeling. American Journal of Physiology. Heart and Circulatory Physiology, 289(5):H2048–2058, November 2005. 16006541 10.1152/ajpheart.00934.2004

[48] Holzapfel Gerhard A., Gasser Thomas C., and Ogden Ray W.. A New Constitutive Framework for Arterial Wall Mechanics and a Comparative Study of Material Models. Journal of Elasticity, 61(1/3):1–48, 2000.

[49] Volokh K.Y.. Modeling failure of soft anisotropic materials with application to arteries. Journal of the Mechanical Behavior of Biomedical Materials, 4(8):1582–1594, November 2011. Publisher: Elsevier BV. 22098860 10.1016/j.jmbbm.2011.01.002

[50] Rosenthal Amnon, Gross Robert E., and Pasternac Andre. Aneurysms of right ventricular outflow patches. The Journal of Thoracic and Cardiovascular Surgery, 63(5):735–740, May 1972. Publisher: Elsevier BV. 5028308

[51] Maas Steve A., Ateshian Gerard A., Weiss Jeffrey A., and Herron Michael. Isotropic Elastic, March 2025.

[52] Sadipour M and Azadani An. The Measurement of Bovine Pericardium Density and Its Implications on Leaflet Stress Distribution in Bioprosthetic Heart Valves. Cardiovascular engineering and technology, 14(6), December 2023. 10.1007/s13239-023-00692-0 37932655

[53] Mc Hsu, Kamensky D, Xu F, Kiendl J, Wang C, Wu Mc, Mineroff J, Reali A, Bazilevs Y, and Sacks Ms. Dynamic and fluid-structure interaction simulations of bioprosthetic heart valves using parametric design with T-splines and Fung-type material models. Computational mechanics, 55(6), June 2015. 10.1007/s00466-015-1166-x PMC4574293 26392645

[54] Bonhoeffer Philipp, Boudjemline Younes, Saliba Zakhia, Hausse Ana Olga, Aggoun Yacine, Bonnet Damien, Sidi Daniel, and Kachaner Jean. Transcatheter Implantation of a Bovine Valve in Pulmonary Position: A Lamb Study. Circulation, 102(7):813–816, August 2000. 10942752 10.1161/01.cir.102.7.813

[55] Zhang X.-Y., Trame M. N., Lesko L. J., and Schmidt S.. Sobol Sensitivity Analysis: A Tool to Guide the Development and Evaluation of Systems Pharmacology Models. CPT: pharmacometrics & systems pharmacology, 4(2):69–79, February 2015. 27548289 10.1002/psp4.6 PMC5006244

[56] Nordmeyer Johannes, Ewert Peter, Gewillig Marc, AlJufan Mansour, Carminati Mario, Kretschmar Oliver, Uebing Anselm, Dähnert Ingo, Röhle Robert, Schneider Heike, Witsenburg Maarten, Benson Lee, Gitter Roland, Bökenkamp Regina, Mahadevan Vaikom, and Berger Felix. Acute and midterm outcomes of the post-approval MELODY Registry: a multicentre registry of transcatheter pulmonary valve implantation. European Heart Journal, 40(27):2255–2264, July 2019. 31005985 10.1093/eurheartj/ehz201

[57] Armstrong Aimee K., Berger Felix, Jones Thomas K., Moore John W., Benson Lee N., Cheatham John P., Turner Daniel R., Rhodes John F., Vincent Julie A., Zellers Thomas, Lung Te-Hsin, Eicken Andreas, and McElhinney Doff B.. Association between patient age at implant and outcomes after transcatheter pulmonary valve replacement in the multicenter Melody valve trials. Catheterization and Cardiovascular Interventions, 94(4):607–617, October 2019. 31419019 10.1002/ccd.28454

[58] Houeijeh Ali, Batteux Clement, Karsenty Clement, Ramdane Nassima, Lecerf Florence, Valdeolmillos Estibaliz, Lourtet-Hascoet Julie, Cohen Sarah, Belli Emre, Petit Jérôme, and Hascoët Sébastien. Long-term outcomes of transcatheter pulmonary valve implantation with melody and SAPIEN valves. International Journal of Cardiology, 370:156–166, January 2023. 36283540 10.1016/j.ijcard.2022.10.141

[59] Wu Wensi, Daneker Mitchell, Herz Christian, Dewey Hannah, Weiss Jeffrey A., Pouch Alison M., Lu Lu, and Jolley Matthew A.. A noninvasive method for determining elastic parameters of valve tissue using physics-informed neural networks. Acta Biomaterialia, 200:283–298, June 2025. Publisher: Elsevier BV. 40436231 10.1016/j.actbio.2025.05.021 PMC12207209

[60] Luraghi Giulia, Migliavacca Francesco, García-González Alberto, Chiastra Claudio, Rossi Alexia, Cao Davide, Stefanini Giulio, and Rodriguez Matas Jose Felix. On the Modeling of Patient-Specific Transcatheter Aortic Valve Replacement: A Fluid–Structure Interaction Approach. Cardiovascular Engineering and Technology, 10(3):437–455, September 2019. 31309527 10.1007/s13239-019-00427-0

[61] Ghosh Ram P., Marom Gil, Bianchi Matteo, D’souza Karl, Zietak Wojtek, and Bluestein Danny. Numerical evaluation of transcatheter aortic valve performance during heart beating and its post-deployment fluid–structure interaction analysis. Biomechanics and Modeling in Mechanobiology, 19(5):1725–1740, October 2020. 32095912 10.1007/s10237-020-01304-9 PMC7483251

[62] Basri Adi A., Zuber Mohammad, Basri Ernnie I., Zakaria Muhammad S., Aziz Ahmad F. A., Tamagawa Masaaki, and Ahmad Kamarul A.. Fluid Structure Interaction on Paravalvular Leakage of Transcatheter Aortic Valve Implantation Related to Aortic Stenosis: A Patient-Specific Case. Computational and Mathematical Methods in Medicine, 2020:1–22, May 2020. 10.1155/2020/9163085 PMC7219000 32454886

[63] Fumagalli Ivan, Polidori Rebecca, Renzi Francesca, Fusini Laura, Quarteroni Alfio, Pontone Gianluca, and Vergara Christian. Fluid-structure interaction analysis of transcatheter aortic valve implantation. International Journal for Numerical Methods in Biomedical Engineering, 39(6):e3704, June 2023. 36971047 10.1002/cnm.3704

